# Exploring the mechanism of SLXG for treating nonalcoholic fatty liver disease based on network pharmacology and molecular docking

**DOI:** 10.1097/MD.0000000000040255

**Published:** 2025-02-07

**Authors:** Yang Wang, Jiaxing Wang, Zitong Chen, Bin Liu, Wujie Wang, Yuliang Li

**Affiliations:** a Department of Intervention Medicine and Microinvasive Oncology, The Second Hospital of Shandong University, Jinan, China; b Institute of Interventional Oncology, Shandong University, Jinan, China.

**Keywords:** molecular docking, NAFLD, network pharmacology, SLXG

## Abstract

**Background:**

The Shugan Lidan Decoction and Chaihu Shugan formula are traditional Chinese medicine formulas for treating liver diseases, with a history of over a 1000 years. By comprehensively improving 2 traditional Chinese medicinal formulas, Shugan Lidan Xiaoshi Granules (SLXG) has been developed for the treatment of nonalcoholic fatty liver disease (NAFLD) and other liver-related metabolic diseases.

**Methods:**

First, the effective active ingredients and targets of SLXG were determined using the Traditional Chinese Medicine Systems Pharmacology Database and Analysis Platform database. The treatment targets for NAFLD were identified using the GeneCards, OMIM, and CTD databases, and the intersection of the decoction and disease targets was obtained. The intersection targets were then subjected to protein–protein interaction network analysis, Kyoto encyclopedia of genes and genomes (KEGG) enrichment analysis, and gene ontology enrichment analysis. KEGG enrichment analysis revealed enrichment of the NAFLD pathway. Molecular docking was performed to validate the binding between the crucial targets enriched in this pathway and the corresponding active ingredients in SLXG.

**Results:**

A total of 219 disease intersection genes related to NAFLD were identified from the GeneCards, OMIM, and CTD databases, and 239 non-duplicated drug targets were obtained from the Traditional Chinese Medicine Systems Pharmacology Database and Analysis Platform database. A total of 24 intersection target genes were obtained from both drug- and disease-related databases, with 6 genes enriched in the KEGG NAFLD pathway. Molecular docking results showed that the 13 gene–active ingredient bindings had a binding energy of less than −6.5.

**Conclusion:**

The use of network pharmacology and molecular docking technology has revealed the mechanism of action of SLXG in NAFLD treatment, thus laying a theoretical foundation for the clinical application of SLXG in NAFLD therapy.

## 1. Introduction

Nonalcoholic fatty liver disease (NAFLD) is a common liver disease that is closely related to factors such as diet and lifestyle.^[1]^ When NAFLD occurs, excessive fat accumulates in the liver tissue, which can lead to impaired liver function and, in severe cases, hepatitis and cirrhosis.^[2]^ The disease typically occurs in populations with risk factors for metabolic syndrome such as obesity, type 2 diabetes, high cholesterol, and high blood pressure.^[3]^ In addition to these factors, genetic predisposition, certain medications, and rapid weight loss may lead to the development of NAFLD.^[4–6]^ In the early stages, NAFLD may not have obvious symptoms; however, as the disease progresses, patients may experience fatigue, abdominal discomfort, jaundice, and other symptoms.^[7]^ Severe NAFLD can result in liver function deterioration, elevating the likelihood of liver complications such as hepatitis, cirrhosis, and liver cancer. Additionally, it may exacerbate conditions in other bodily systems, including cardiovascular disease and metabolic syndrome.^[8]^ Clinical risks are associated with cognitive decline and mental illness.^[9]^ A significant correlation exists between NAFLD and cognitive deficits, increasing the risk of neurodegenerative disorders such as dementia. This association is presumably due to common pathophysiological factors, including insulin resistance, inflammation, and lipid metabolism dysregulation.^[10]^ Moreover, the prevalence of mental health disorders, such as depression and anxiety, is higher among individuals with NAFLD, complicating the management of this liver condition.^[11]^ Considering these complexities, the management of NAFLD extends beyond treating the liver condition itself to address the associated systemic and psychiatric complications. This comprehensive management strategy involves lifestyle modifications, specific pharmacological treatments, and potentially mental health support or psychiatric interventions for those suffering from concurrent mental health issues.^[11]^ Consequently, NAFLD poses a significant threat to patient health.

Traditional Chinese medicine (TCM) is a therapeutic practice originating from ancient medicine in China, with a history spanning thousands of years.^[12]^ TCM is primarily derived from botanical, zoological, and mineral sources, and is commonly administered as an herbal remedy. It is extensively used in the prevention and treatment of diverse illnesses and has significant relevance in clinical settings. TCM is frequently prepared as herbal materials, pills, decoctions, and granules and is renowned for its natural, gentle, and comprehensive regulatory attributes. Evidence from recent studies has underscored the potential of TCM in addressing NAFLD. Investigations have highlighted the ability of the herbal concoction Salvia-Nelumbinis naturalis to influence various biological pathways. This extract shows particular efficacy in regulating the SIRT1/AMPK pathway, which is vital for cellular energy regulation and lipid metabolism, thus facilitating lipid metabolic improvements in NAFLD.^[13]^ Moreover, a range of natural small-molecule medications have demonstrated the ability to mitigate fibrosis, oxidation, and inflammation by acting on critical signaling pathways pivotal in NAFLD progression.^[13]^ In addition, this study utilized network pharmacology to merge empirical evidence with molecular biology, shedding light on the intricate interactions between TCM’s active components of TCM and biological targets related to NAFLD.^[14]^ This methodology not only validates the effectiveness of traditional remedies but also deepens the understanding of their mechanisms, thereby enhancing clinical practices and fostering the innovation of novel therapeutic approaches. The Shugan Lidan Decoction and Chaihu Shugan formulas are TCM compound formulas that have been utilized for the treatment of NAFLD.^[15,16]^ We made comprehensive enhancements to these 2 compound formulas to develop Shugan Lidan Xiaoshi Granules (SLXG), which is intended for the treatment of NAFLD and other liver metabolic diseases. Due to the unclear mechanism of action and unidentified targets of TCM compound formulas, it is essential to study the pharmacological effects of TCM compound formulas. This will provide an in-depth understanding of the mechanism of action and efficacy of the SLXG compound formula in the treatment of NAFLD, providing a scientific basis for its clinical application.

In recent years, network pharmacology has been widely applied in drug development. It utilizes techniques such as bioinformatics and network analysis to reveal the multiple active components and targets of drugs in an organism, providing theoretical guidance for drug design and optimization. Network pharmacology methods are valuable for identifying the active components and targets of action in TCM formulas. Analyzing the interactions between drug molecules and targets aids in uncovering the active ingredients and pharmacological mechanisms of TCM formulas. Utilizing this method enables a comprehensive understanding of the therapeutic components of TCM formulas, offering new pathways and perspectives for TCM pharmacological research.^[17]^ Molecular docking is a simulation technique used to predict the binding mode between proteins and small molecules. After constructing a network of active ingredients and targets through network pharmacology, the likelihood of the binding of active ingredients to targets was simulated by molecular docking, and the interaction between the 2 was determined. This provides strong evidence for the target of action of TCM formulas.^[18]^ This allows a more nuanced understanding of the therapeutic effects of TCM formulations at the molecular level. By predicting and visualizing the interactions between the active ingredients and therapeutic targets, we can potentially uncover new therapeutic targets. Furthermore, this method facilitates the optimization of TCM formulations by identifying the most effective compounds, thus enhancing the efficacy and specificity of the treatments. Molecular docking serves as a bridge between traditional medicinal knowledge and modern scientific validation, providing a compelling tool for the modernization and globalization of TCM. This integration of techniques fortifies the credibility of TCM in the scientific community, paving the way for its broader acceptance and integration into conventional medical frameworks.

## 2. Materials and methods

### 2.1. Prediction of the active constituents and target proteins

In the exploration of SLXG, a TCM formulation, this study employed the Traditional Chinese Medicine Systems Pharmacology Database and Analysis Platform (TCMSP, accessible at TCMSP - Traditional Chinese Medicine Systems Pharmacology Database and Analysis Platform) to identify essential active components. This platform facilitated the precise extraction of key active substances from 11 distinct herbs, each of which exhibited potential pharmacological effects. Initially, these substances underwent assessments of drug-likeness and bioavailability; components achieving a drug-likeness index of no <0.18 and bioavailability exceeding 30% were deemed effective. These stringent criteria guaranteed the effective absorption of the selected compounds and confirmed their practicality as oral medications. The retrieval of target proteins for these efficacious components provided critical evidence for the potential therapeutic mechanisms of SLXG.

### 2.2. Identification of targets associated with the disease

Within the Genecard database (https://www.genecards.org/),^[19]^ a comprehensive search was performed using “nonalcoholic fatty liver disease” as the keyword. Genes listed in the results with a relevance score >1 were flagged as potential disease-related targets. This relevance score is calculated by Genecard based on the association of the gene with the disease, where a higher score indicates a stronger association. A similar keyword search was conducted using the CTD database (http://ctdbase.org/),^[20]^ a similar keyword search was conducted. From the retrieved gene list, genes with an inference score of 20 or higher were selected as disease-related targets. The inference score in the CTD quantifies the strength of the evidence supporting the gene–disease association derived from various sources, including experimental and curated databases. The OMIM database (https://www.omim.org/)^[21]^ was queried using the same keywords. This database, which focuses on genetic disorders and phenotypes, provides a list of genes associated with NAFLD based on the literature and annotated research studies. No specific scoring threshold was used for OMIM, and all genes directly mentioned in the context of NAFLD were considered relevant. After collating the gene lists from all 3 databases, an upset plot was generated to visualize the overlap of the gene lists. The Upset plot, created using the UpsetR package in R, provides a detailed view of the intersection and unique genes of each database. Genes appearing in at least 2 databases were designated as disease-related genes for NAFLD.

### 2.3. Exploration of pivotal therapeutic targets

First, 2 separate datasets were imported into the R programming environment: 1 containing genes associated with specific diseases, and the other containing genes targeted by various drugs. After importing these datasets, the ggvenn package in R was used to construct a Venn diagram. This diagram visually displays the overlapping and unique genes of the 2 gene sets, allowing for easy comparison. Finally, the genes that appeared in both datasets were identified and exported into a separate file for further analysis.

### 2.4. Establishing a network pharmacology network

Begin identifying the active ingredients, as predicted by TCMSP, and their corresponding targets. The next step was to import this dataset into Cytoscape software version 3.9.1. Within Cytoscape, we used these data to construct a comprehensive pharmacology network that visually represents the interactions between these active ingredients and their gene targets. This network can then be analyzed to explore the potential therapeutic pathways and mechanisms of action inherent in TCM.

### 2.5. KEGG enrichment analysis and GO enrichment analysis

Begin importing the list of intersecting genes into the R software. With these genes loaded, the clusterProfiler package was used to conduct a thorough enrichment analysis. This analysis should encompass both the Kyoto encyclopedia of genes and genomes (KEGG) pathway and gene ontology (GO) enrichment. GO enrichment analysis included all 3 major categories: biological process, cellular component, and molecular function. Once the enrichment analysis was completed, GraphPad Prism (version 9.0) was used to visualize the results. This visualization should include detailed and clear enriched pathways and GO terms to help elucidate the biological significance, underlying pathways, and functional roles of the intersecting genes. This comprehensive approach provides a deeper understanding of the involvement of genes in various biological processes and their potential implications in disease and drug interactions.

### 2.6. Protein–protein interaction (PPI) network

Begin to import a list of 24 genes, identified as intersection genes from the previous analysis, into the STRING database. This platform was designed to analyze known and predicted PPI. Once the genes are uploaded, STRING tools are utilized to construct a PPI network based on these genes. After the PPI network was generated, the results were exported from the STRING database. Next, we used the Cytoscape software, an open-source platform for complex network analysis and visualization, to create a diagram of the PPI network. This visualization will help in understanding the interplay between proteins and may reveal key hubs and signaling pathways within the network.

### 2.7. Molecular docking

#### 2.7.1. Preparation and preprocessing

The PDB database (https://www1.rcsb.org/) was used to search for and retrieve the three-dimensional structures of the target proteins ADIPOQ (PDB ID: 6U66), PPARA (PDB ID: 2P54), IL6 (PDB ID: 1AIU), CXCL8 (PDB ID: 6N2U), AKT1 (PDB ID: 6S9W), and ACACA (PDB ID: 6G2D). The molecular structure file of SREBF1, predicted by Alphafold with high confidence, was obtained from the UniProt database for docking purposes. The molecular structure files of the ligands acacetin, beta-carotene, kaempferol, luteolin, naringenin, paeoniflorin, and quercetin were obtained from the PubChem database. Utilize PyMOL 2.3.0 software to conduct operations such as removing water molecules and original ligands from the retrieved target proteins. Employ Chem3D (2020 version) software for the molecular mechanics optimization of small molecules to achieve energetically minimized optimal conformations. Auto Dock Tools 1.5.6 for preprocessing the target proteins and ligands to obtain pdbqt files. Molecular simulation docking of the target proteins and ligands was performed using Auto Dock Vina v.1.2.0 software. The docking algorithm employed was the Lamarckian genetic algorithm, with semi-flexible docking, exhaustiveness set to 8, and the maximum number of output conformations set to 9.

## 3. Results

### 3.1. Identification of active constituents and therapeutic targets of SLXG

*Glycyrrhiza uralensis*, Flos Lonicerae, Christina Loosestrife Herb, Radix Bupleuri, Immature Trifoliate-orange Fruit, Chuanxiong Rhizoma, Japanese Climbing Fern Spore, Herba Artemisiae Scopariae, white paeony root, and turmeric root tubers in SLXG were used to search for active ingredients in the TCMSP database. This search resulted in the discovery of 140 active ingredients with a drug-likeness of ≥ 0.18 and bioavailability of ≥ 30%. In addition, 239 therapeutic targets associated with these active ingredients were identified. Target genes corresponding to these active ingredients were converted into human gene names using the UniProt database. Finally, a table corresponding to TCM, active ingredients, and therapeutic targets was obtained through the organization (Table S1, Supplemental Digital Content, http://links.lww.com/MD/O304).

### 3.2. Search for NAFLD-related genes

**Figure 1.**
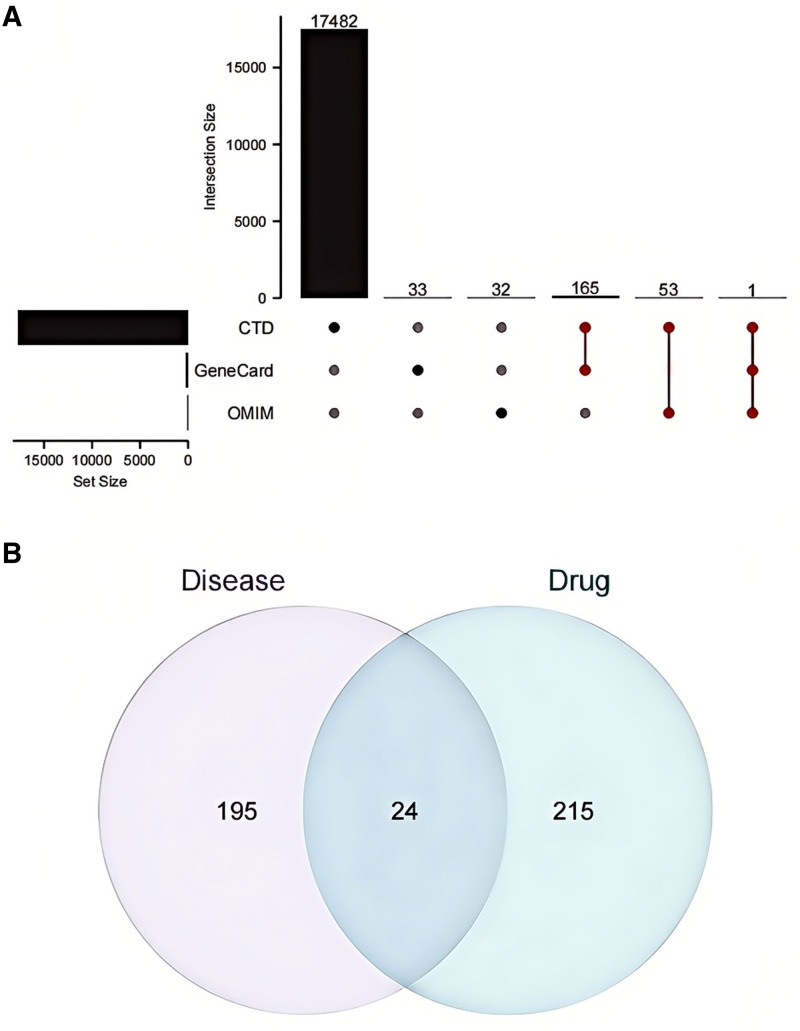
Identification of therapeutic targets for SLXG. (A) Searching for disease-related targets; (B) exploration of disease-specific therapeutic targets associated with traditional Chinese herbal compound formulations. SLXG = Shugan Lidan Xiaoshi Granules.

Using NAFLD as a search term, disease-related genes were retrieved from the CTD, GeneCard, and OMIM databases. Subsequently, it was determined that there were 17,701, 109, and 86 target genes in CTD, GeneCard, and OMIM, respectively. Utilizing the Upset plot to illustrate the distribution of target genes across these databases, we found that there were 17,482 unique target genes in CTD, 33 in Genecard, and 32 in OMIM. Furthermore, there were 165 target genes common to both CTD and Genecard, 53 target genes common to both CTD and OMIM, and only 1 target gene present in all 3 databases (CTD, Genecard, and OMIM). The target genes present in 2 or more databases were identified as disease-related therapeutic targets, totaling 219 genes (Fig. 1A, Table S2, Supplemental Digital Content, http://links.lww.com/MD/O304).

### 3.3. Identification of drug–disease intersection targets

To identify vital therapeutic targets, we conducted a comparative analysis of the action targets of SLXG with those related to diseases and illustrated the findings through a Venn diagram. Among these, 215 genes were associated with drug-related targets, 195 were associated with disease-related targets, and 24 were associated with drug–disease intersection targets (Fig. 1B, Tables S3, Supplemental Digital Content, http://links.lww.com/MD/O304).

### 3.4. Constructing the pharmacological network of TCM–active ingredient–therapy targets

**Figure 2.**
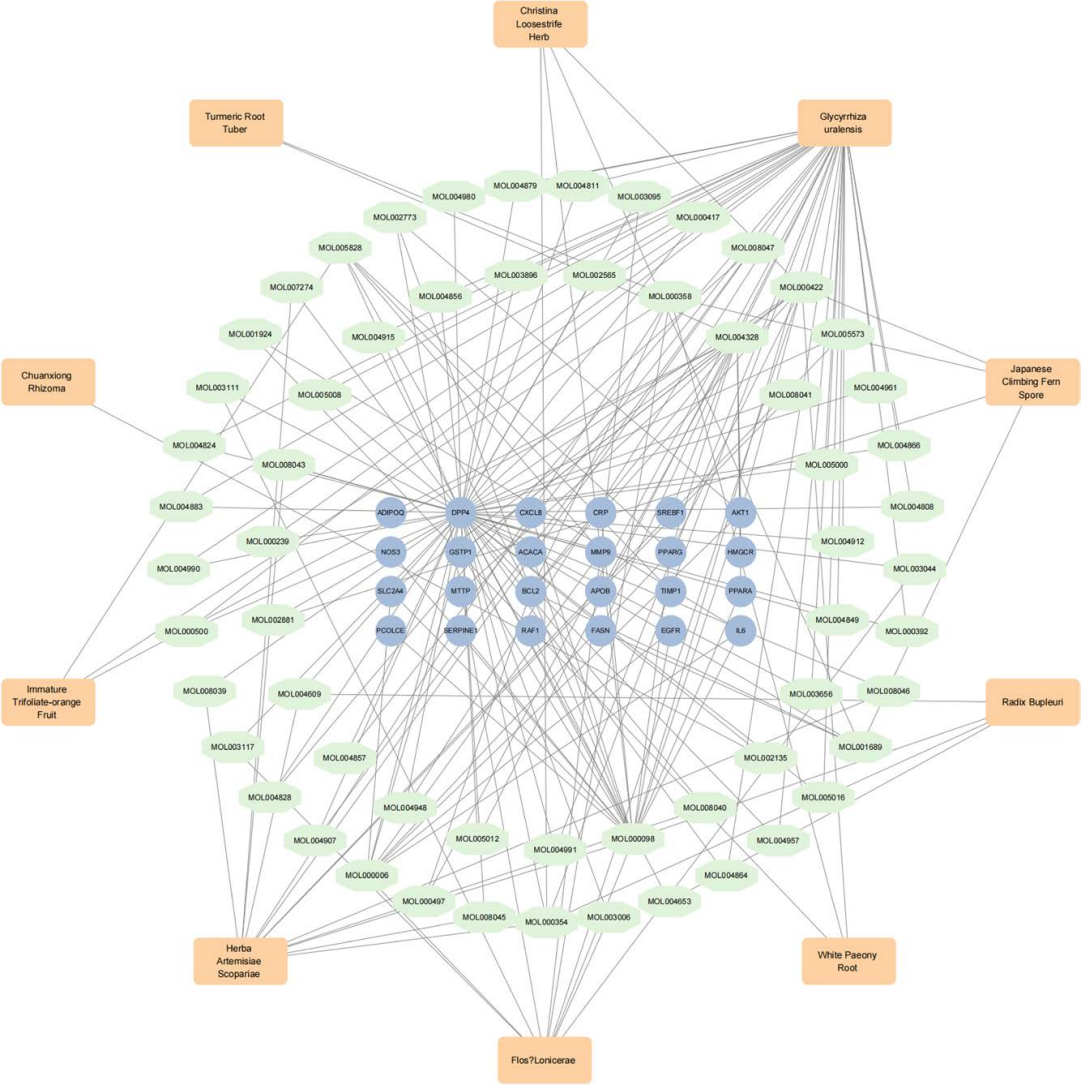
Constructing the pharmacological network of traditional Chinese medicine–active ingredient–therapy targets.

A network of drugs, active ingredients, and targets was constructed using the Cytoscape software. The network comprised 24 targets, 60 active ingredients, and 10 Chinese herbal medicines. Therapeutic targets are represented by blue circular icons arranged in the middle as a square, active drug ingredients are denoted by green octagonal icons arranged in a ring, and Chinese herbal medicines are depicted as the outermost orange square icons. *G uralensis* was associated with the highest number of active ingredients in the network, potentially playing a crucial therapeutic role in the treatment of NAFLD (Fig. 2, Table S4, Supplemental Digital Content, http://links.lww.com/MD/O304).

### 3.5. GO and KEGG enrichment analysis

**Figure 3.**
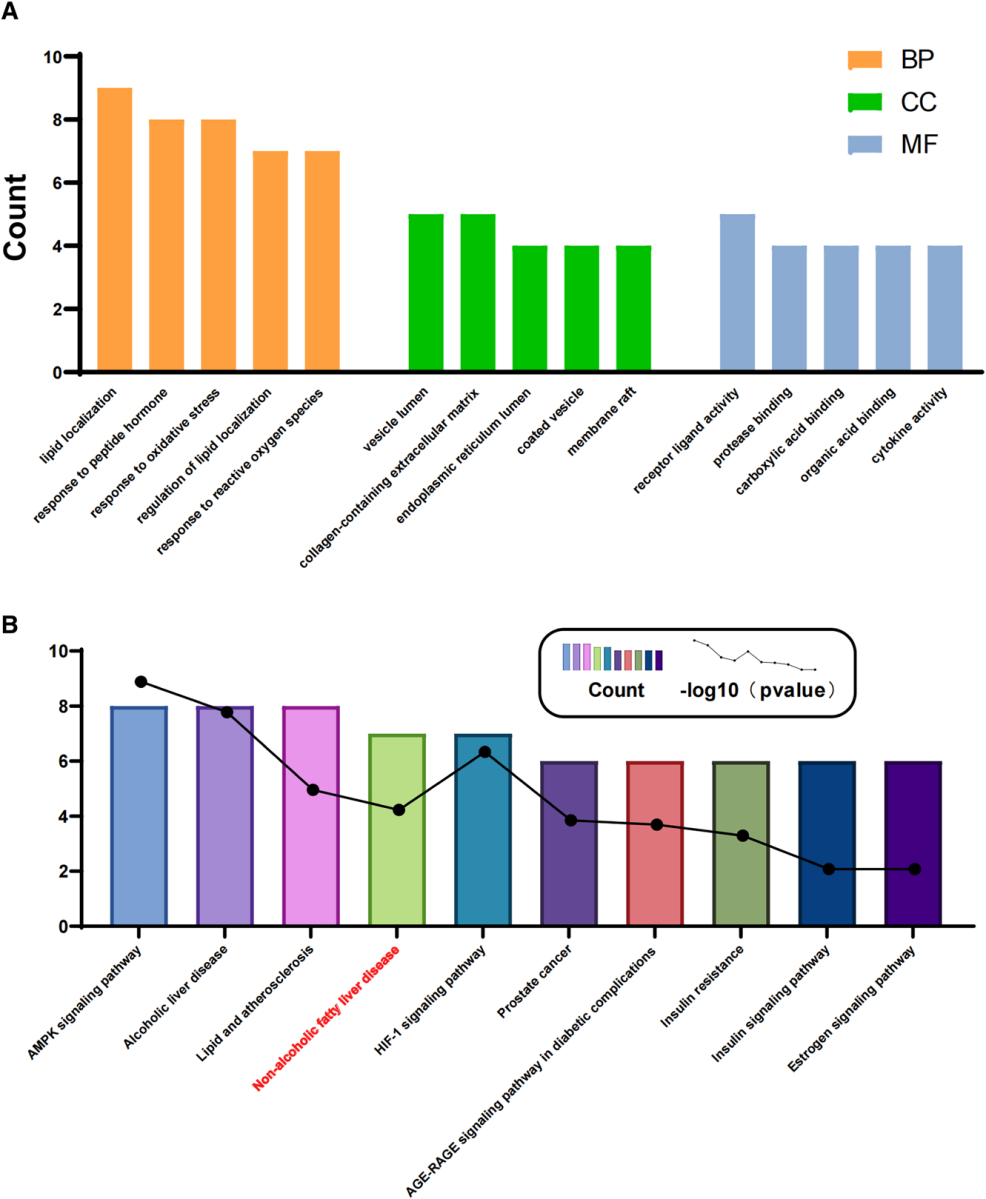
GO and KEGG enrichment analysis. (a) GO enrichment analysis including biological process (BP), cellular component (CC), and molecular function (MF); (b) KEGG enrichment analysis. GO = gene ontology, KEGG = Kyoto encyclopedia of genes and genomes.

Using the 24 therapeutic targets, we conducted GO and KEGG enrichment analyses. The GO analysis results revealed that biological processes were predominantly enriched in lipid localization, response to peptide hormone, response to oxidative stress, regulation of lipid localization, and response to reactive oxygen species; cellular components were mainly enriched in the vesicle lumen, collagen-containing extracellular matrix, endoplasmic reticulum lumen, coated vesicle, and membrane raft; and molecular functions were principally enriched in receptor ligand activity, protease binding, carboxylic acid binding, organic acid binding, and cytokine activity (Fig. 3A). These findings suggest that SLXG could potentially address NAFLD by modulating lipid transport and metabolism, oxidative stress, and binding of organic acids, including bile acids. KEGG enrichment analysis revealed an enrichment of the NAFLD pathway, suggesting that SLXG may potentially treat nonalcoholic steatohepatitis (NASH) by regulating the expression of genes such as CXCL8, AKT1, SREBF1, IL6, PPARA, and ADIPOQ (Fig. 3B).

### 3.6. Constructing a PPI network

**Figure 4.**
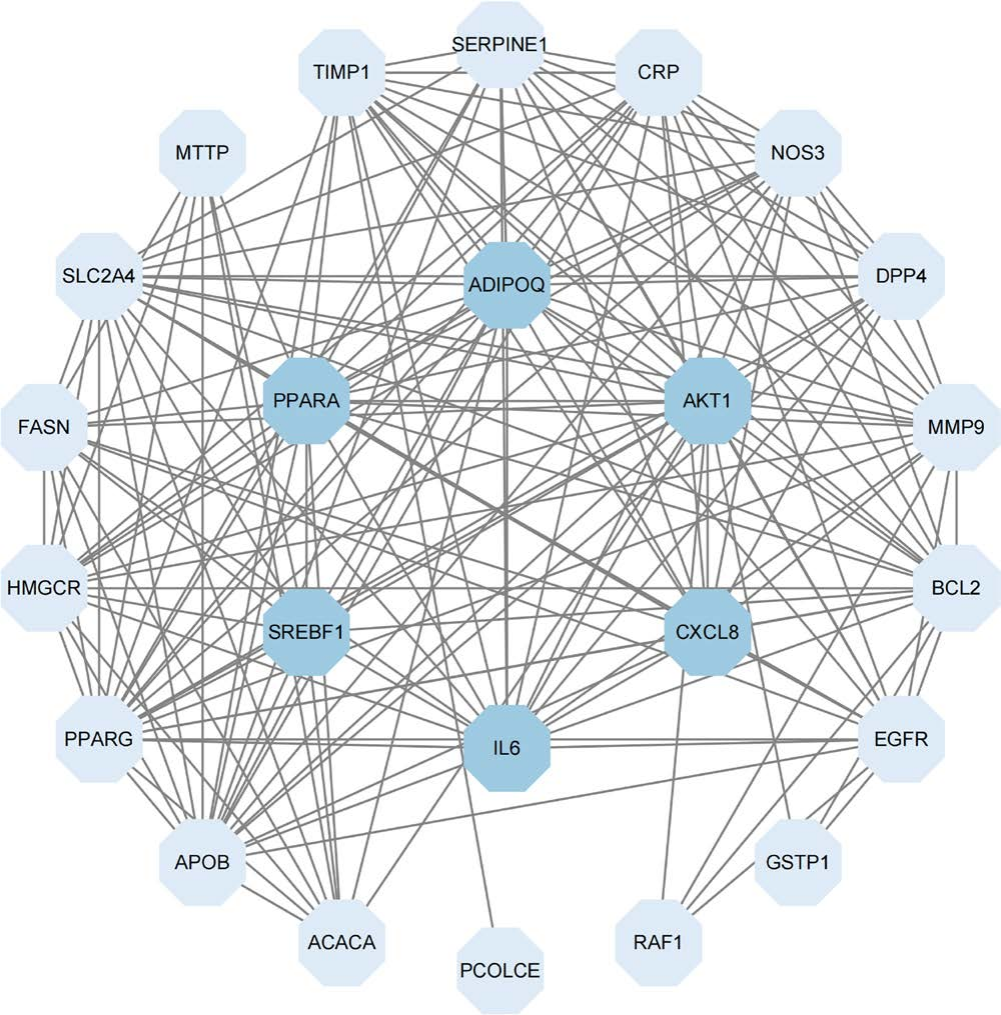
PPI network of intersection target. PPI = protein–protein interactions.

The 24 intersecting genes were uploaded to the STRING website, and a protein interaction network was constructed using the Cytoscape software. In the network representation, octagons symbolize proteins, with darker ones in the center representing genes enriched in the KEGG pathway analysis of NAFLD, while the light blue ones on the periphery represent genes not enriched in the drug–disease intersection (Fig. 4).

### 3.7. Molecular docking results

**Figure 5.**
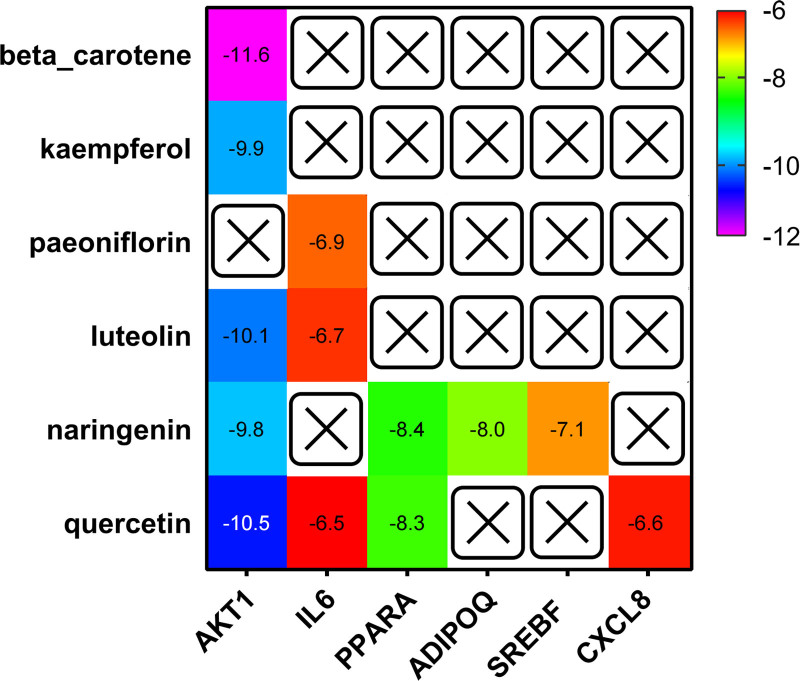
Heatmap of binding affinity between active compounds in traditional Chinese medicine and therapeutic target molecules.

**Figure 6.**
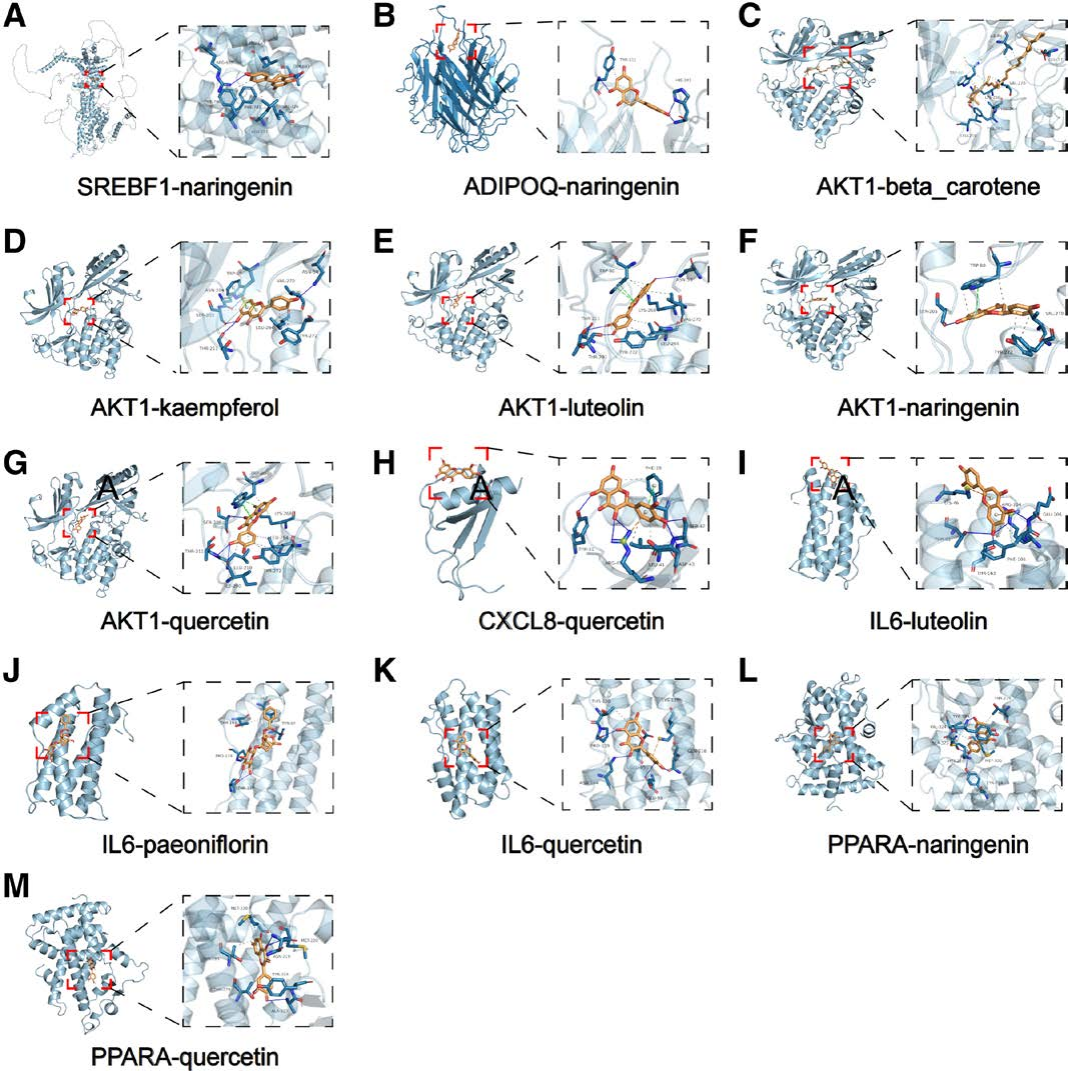
The molecular docking of active components with binding energies less than −6.5 with therapeutic targets.

Genes enriched in the NAFLD pathway in KEGG were docked with their corresponding active ingredients (Fig. 5). The molecular docking results showed that there were 12 binding energies less than −6.5, among which 8 showed strong binding with energies less than −7.5. Five active compounds were bound to AKT1 and all exhibited binding energies below −9.5. Three active compounds bind to IL6, with paeoniflorin and naringenin showing binding energies with IL6 lower than −6.5. Quercetin and naringenin bind PPARA, and their binding energies are less than −7.5. ADIPOQ and SREBF bind naringenin, whereas CXCL8 binds quercetin. Each of these 3 targets binds to only 1 active ingredient. Figure 6 shows the 3D molecular docking images of proteins and active ingredients with binding energies less than −6.5 (Fig. 6).

### 3.8. The mechanism pathway diagram of SLXG in the treatment of NAFLD

**Figure 7.**
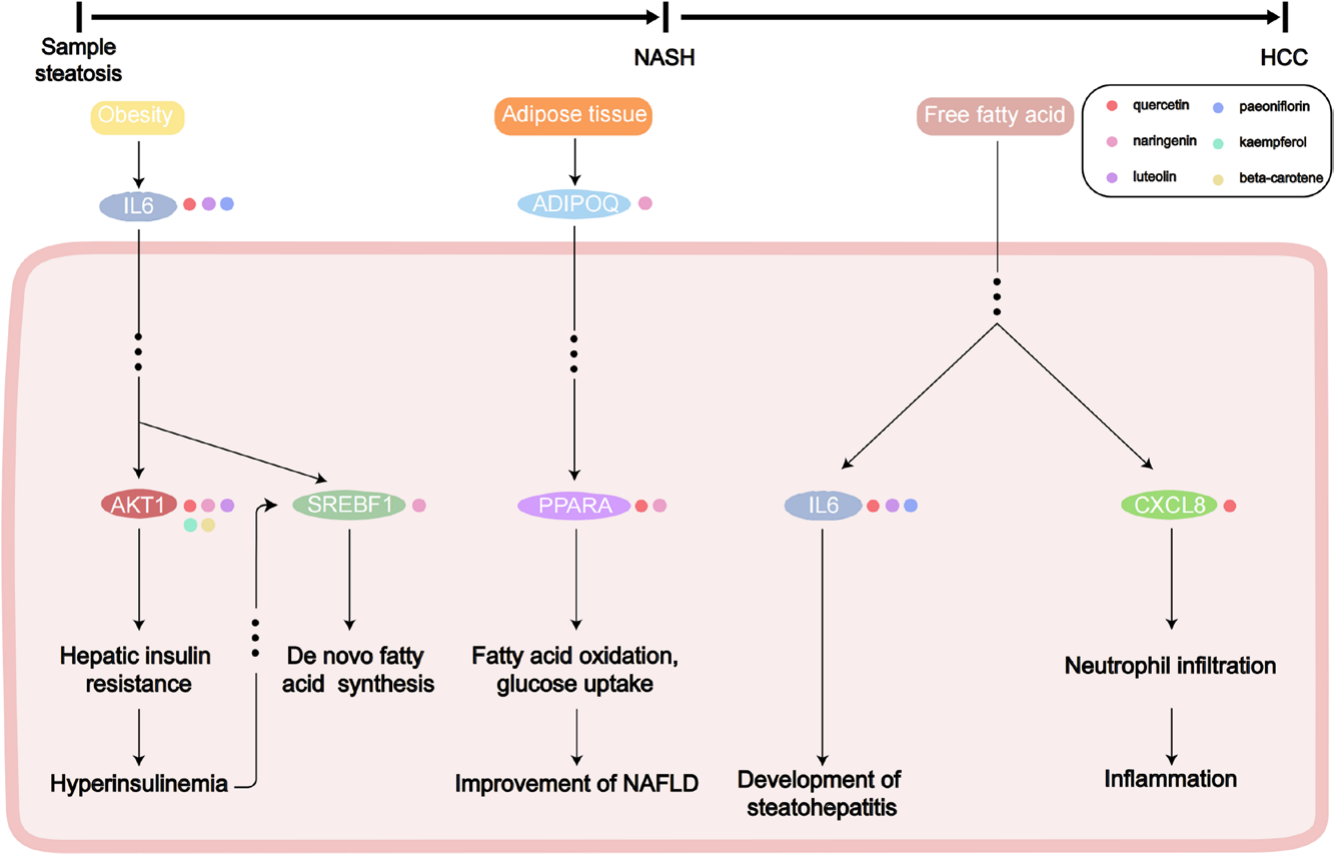
Molecular mechanism diagram of SLXG in treating NAFLD. NASH = nonalcoholic steatohepatitis, SLXG = Shugan Lidan Xiaoshi Granules.

The mechanistic diagram presented in this study is based on the NAFLD pathway identified in KEGG and the active ingredients in SLXG, along with their corresponding targets. In the initial stages of NAFLD, hepatic cell steatosis is the primary manifestation is hepatic cell steatosis. Obesity indirectly regulates de novo lipogenesis of fatty acids by controlling downstream AKT1 and SREBF1 through IL6. During progression to NASH, adipose tissues modulate the downstream PPAR-α through ADIPOQ, enhancing the oxidation of fatty acids and glucose uptake in NAFLD. As the disease progresses from NASH to hepatocellular carcinoma, liver inflammation becomes a significant factor. Fatty acids in the liver promote the advancement of fatty liver inflammation through IL6 and intensify neutrophil infiltration to stimulate inflammation development via IL8. The 6 active ingredients in SLXG, quercetin, naringenin, luteolin, paeoniflorin, kaempferol, and beta-carotene, may influence the progression of NAFLD at different stages by acting on 1 or more of the aforementioned targets, thereby achieving a good therapeutic effect on NAFLD (Fig. 7).

## 4. Discussion

NAFLD is a liver disease caused by nonalcohol-related metabolic disorders of fat and has become one of the most common chronic liver diseases worldwide.^[1]^ TCM boasts a rich historical legacy in China and has been employed for millennia in the treatment of ailments and preservation of well-being. Chinese herbal medicines are extracted from natural sources, including plants, animals, and minerals, and have widespread applications in TCM practice. The theoretical framework of TCM emphasizes equilibrium and holistic health, and perceives the body as an integrated system. Consequently, Chinese herbal medicines are frequently employed in compound formulations for disease management and the regulation of bodily functions. The Shugan Lidan Decoction and Chaihu Shugan formula are TCM formulas for treating liver diseases, with a history of over a 1000 years. The Shugan Lidan Decoction treats NAFLD by upregulating the expression of serum adiponectin (ANA) and downregulating ferritin (SF) in patients. Previous studies have reported that the Chaihu Shugan formula can improve lipid disorders in the liver of rats, slow down the progression of NASH, and mediate anti-inflammatory effects in rat liver cells by regulating the p38–MAPK pathway.^[22]^ The Chaihu Shugan formula can also regulate gut microbiota imbalance in NAFLD rats through NLRP3 and alleviate chronic metabolic inflammation.^[15]^ Additionally, the Chaihu Shugan formula has been reported to have a protective effect in insulin-resistant rats with nonalcoholic fatty liver.^[16]^ By comprehensively improving 2 traditional Chinese medicinal formulas, SLXG has been developed for the treatment of NAFLD and other liver-related metabolic diseases.

NAFLD development is closely linked to lipid synthesis, oxidative stress, and bile acid synthesis. Prior research has demonstrated that loquat fruit extract containing polyphenols (LFP) mitigates abnormal weight, lipid metabolism disorders, oxidative stress, and inflammation.^[23]^ Lactobacillus NKK20 ameliorates NAFLD in mice by modulating bile acid synthesis metabolism.^[24]^ Enrichment analysis using GO indicated that SLXG may effectively treat NAFLD by regulating lipid transport and metabolism, oxidative stress, and binding of organic acids, including bile acids. Furthermore, KEGG analysis identified enrichment of the NAFLD pathway, and the roles of enriched genes in NAFLD development were elucidated through a pathway diagram (see Fig. 7). During the early stages of NAFLD, obesity regulates fatty acid synthesis via the IL6/AKT/SREBP1c pathway. In the NASH stage, adipose tissue enhances liver fatty acid oxidation and glucose uptake by modulating the ADIPOQ/PPARA pathway. As NASH progresses to hepatocellular carcinoma, free fatty acids exacerbate liver inflammation via the pro-inflammatory factors IL6 and IL8. Previous studies have demonstrated that polysaccharides derived from tartary buckwheat roots can improve NAFLD through the IL6/SOCS3/SREBP1c pathway.^[25]^ The ADIPOQ receptor family member, PAQR9, can influence fasting-induced hepatic ketogenesis and fatty acid oxidation by regulating PPARα.^[26]^ Additionally, IL8 shows a significant correlation with transaminase levels and histological severity of NALFD,^[27]^ with liver IL6 expression levels being higher in NAFLD patients than in patients with simple hepatic steatosis.^[28]^

This study highlights the unique advantages of TCM in treating NAFLD, offering new directions for the treatment of NAFLD and related metabolic diseases through the development of improved TCM formulas, namely SLXG. Additionally, potential molecular mechanisms of SLXG in treating NAFLD have been revealed through GO and KEGG analyses, emphasizing the importance of studying the molecular mechanisms in TCM treatments. This research is innovative and forward-looking for exploring TCM treatments for NAFLD, promising to provide valuable guidance and insights for further studies in this field. Moreover, in contrast to the existing treatment methods, this study highlights the unique advantages and potential contributions of TCM, offering diverse treatment options to the medical community. With the ongoing development and deepening research into TCM, we are confident of its potential for treating NAFLD and look forward to the emergence of more innovative results that could provide more effective treatment options for a broad range of NAFLD patients. Despite significant progress in exploring TCM for the treatment of NAFLD, some profound shortcomings warrant further consideration. First, although network pharmacology and molecular docking provide a systematic research approach, these results still require validation in animal models and clinical trials to ensure their reliability and clinical applicability. Additionally, while the potential mechanisms of SLXG in NAFLD have been mentioned, a deeper understanding of its mechanisms at different pathological stages, potential side effects, and safety concerns is required. Considering the diversity and complexity of NAFLD etiology, future studies should focus on exploring personalized treatment strategies to better meet the needs of different patients. Moreover, more high-quality clinical trials are needed to evaluate the long-term efficacy and safety of TCM in treating NAFLD, and to offer more reliable treatment options for patients. Although this study provides valuable insights for subsequent research, we must continue to address these challenges in future studies to develop and apply more effective NAFLD treatment strategies.

In addition to the current shortcomings, some future prospects are worth our attention. First, as we delve deeper into the mechanisms of TCM treatment for NAFLD, we hope to discover more effective drug targets and treatment strategies, thus providing more options for patients with NAFLD. Second, by integrating the concepts of personalized and precision medicine, future research could explore the possibility of individualized treatment based on patient genotypes, phenotypes, and lifestyle factors to enhance treatment effectiveness and reduce side effects. Moreover, conducting more clinical studies that combine the strengths of TCM and modern medicine to validate the efficacy and safety of TCM in treating NAFLD will provide more evidence to support its clinical application. Finally, enhancing interdisciplinary cooperation to promote the integration of Chinese and Western medicine could offer more comprehensive and effective solutions for the integrated treatment of NAFLD. In future research, deep learning can be utilized to enhance the efficiency and accuracy of predicting potential drug candidates; apply quantum mechanics for in-depth analysis of molecular interactions; and incorporate Absorption, Distribution, Metabolism, and Excretion properties to evaluate drug-likeness at the early stages of the drug discovery process.^[29]^ Therefore, despite existing challenges and unknowns, we are confident of the potential of TCM in treating NAFLD and look forward to future research achieving greater breakthroughs and progress.

This study utilized network pharmacology and molecular docking techniques to explore the active constituents and therapeutic targets of SLXG in the treatment of NAFLD. Through the analysis of PPI networks, enrichment of the biological functions of graphene oxide, and KEGG pathway analysis, the study investigated the crosstalk between disease and drug targets. This study identified genes enriched in the NAFLD pathway through KEGG analysis and conducted molecular docking of these genes with their respective active constituents. Docking results with a score lower than −6.5 were deemed indicative of potential interactions. Subsequently, 13 pairs of drug–protein binding with potential interactions were successfully identified. Furthermore, these findings were used to construct a mechanistic diagram of SLXG for the treatment of NAFLD. This study offers valuable insights and directions for future NAFLD treatment, potentially expanding the treatment options for patients and having a promising impact on clinical applications.

## 5. Conclusion

This study utilized network pharmacology and molecular docking to preliminarily predict the principal active ingredients, targets, and pathways of SLXG for the treatment of NAFLD. Six active ingredients were identified that primarily targeted AKT1, IL6, PPARA, ADIPOQ, SREBF, and CXCL8 in the NAFLD pathway. The findings of this study require further validation through further experiments. Consequently, this study offers a foundational framework for future research on the fundamental mechanisms of SLXG in NAFLD treatment.

## Author contributions

**Conceptualization:** Yang Wang.

**Data curation:** Yang Wang, Jiaxing Wang.

**Funding acquisition:** Bin Liu, Yuliang Li.

**Investigation:** Yang Wang, Jiaxing Wang, Wujie Wang.

**Methodology:** Yang Wang, Zitong Chen, Yuliang Li.

**Project administration:** Wujie Wang, Yuliang Li.

**Validation:** Yang Wang, Jiaxing Wang.

**Writing – original draft:** Yang Wang, Jiaxing Wang, Zitong Chen, Wujie Wang.

**Writing – review & editing:** Bin Liu, Wujie Wang, Yuliang Li.

## Supplementary Material


